# Current indications for acute total hip arthroplasty in older patients with acetabular fracture: Evidence in 601 patients from 2002 to 2021

**DOI:** 10.3389/fsurg.2022.1063469

**Published:** 2023-01-06

**Authors:** Bin-Fei Zhang, Yan Zhuang, Lin Liu, Ke Xu, Hu Wang, Bo Wang, Hong-Quan Wen, Peng Xu

**Affiliations:** ^1^ Department of Joint Surgery, Honghui Hospital, Xi’an Jiaotong University, Xi'an, China; ^2^ Department of Orthopaedic Trauma, Honghui Hospital, Xi’an Jiaotong University, Xi'an, China

**Keywords:** acute total hip arthroplasty, acetabular fracture, geriatric patients, systematic review, indications

## Abstract

**Purpose:**

Older patient population with acetabular fractures is increasing rapidly, requiring enhanced recovery. Acute total hip arthroplasty (THA) is a good option for these patients, and it is becoming increasing popular. However, acute THA has different indications in different studies. Therefore, a systematic review is needed to assess and comprehend the indications for acute THA in older patients.

**Methods:**

A systematic literature review was conducted to identify a retrospective series or prospective studies in older patients (>60 years) with acetabular fractures. The search timeline was from database construction till December 2021; PubMed, Embase, and Cochrane Library databases were searched. Two trained professional reviewers independently read the full text of documents that met the inclusion criteria and extracted information on the specific methods used and indication information based on the research design.

**Results:**

In total, there were 601 patients with acetabular fractures aged >60 years from 33 studies were obtained. Twenty-eight studies reported that THA was a feasible treatment option for acetabular fractures in geriatric patients with good outcome. The primary indications were dome impaction, irreducible articular comminution, femoral head injury, and pre-existing osteoarthritis or avascular necrosis. The most common patterns were anterior column and posterior hemitransverse, posterior wall, both columns, and T-type.

**Conclusion:**

Acute THA is an effective treatment strategy for older patients with acetabular fractures and should be considered when the abovementioned indications are observed on preoperative images. (PROSPERO: CRD42022329555).

## Introduction

Due to the ongoing demographic change, the incidence of acetabular fractures in patients aged >60 years has markedly increased (1, 2). Herath et al. reported that >50% of patients with acetabular fractures were aged ≥60 years, with the oldest reported patient being 80 years old from the German multicenter pelvic registry system (2). Indeed, geriatric patients constitute the most rapidly growing subgroup of acetabular fractures.

For geriatric patients with acetabular fractures, treatment objectives are rapid mobilization of patients on walkers or crutches (3) and rapid recovery to pre-injury level of function (4, 5). Open reduction with internal fixation (ORIF) used to be the mainstay of surgical treatment. However, ORIF in older patients is technically and strategically challenging, with high failure rates after ORIF. A retrospective study reported a 30% failure rate (6). Another study on even older patients reported a failure rate of 45% in ORIF cases (7). With the development of surgical equipment, instruments, and technology, treatment for acetabular fracture has considerably changed. Therefore, some authors believe that acute total hip arthroplasty (THA) may be more beneficial than ORIF and should be strongly considered in older patients with acetabular fractures (8, 9).

Acute THA for displaced acetabular fractures was first reported by Westerborn in 1954 (10). Theoretically, by allowing immediate weight bearing and faster rehabilitation, THA could reduce the risks of early and late local complications associated with the ORIF for this injury type (11). However, indications for acute THA are different across studies. Anglen et al. reported dome impaction as an indication of failure for the internal fixation of acetabular fractures in geriatric patients (12). Kreder et al. reported that marginal impaction and residual displacement of >2 mm were associated with the development of arthritis, which was related to poor function and THA requirement (13). Solomon et al. reported that a displaced fracture involving the anterior or both columns, irreducible articular comminution, protrusion of >1 cm, and osteoporosis were indications for immediate THA (14). In addition, the fracture pattern is another factor that needs consideration in THA treatment. Aprato et al. (15) reported that fractures of the posterior column and/or wall with severe cartilage damage can be treated safely with acute THA. In Borg et al. (16), more patients had anterior column and posterior hemitransverse and both column patterns. In Sarantis et al. (17), the surgeons preferred THA for patients with anterior column to other pattern.

For these reasons, a systematic evaluation system needs to be established to evaluate and comprehend the indications for acute THA in older patients with acetabular fractures. In this systematic review, we aimed to summarize the individual indications and fracture patterns from case series of acute THA for acetabular fractures in older patients.

## Materials and methods

### Inclusion criteria

The ethical approval of the systematic review was waived by the institutional review board of Honghui Hospital, Xi'an Jiaotong University. The inclusion criteria were as follows: 1) **Research type**, retrospective or prospective series studies; 2) **Research participants**, older patients (≥60 years) with unilateral or bilateral acetabular fractures who underwent acute THA; 3) **Index of interest**, factors and indications for acute THA in these patients. The exclusion criteria were as follows: 1) age <60 years, staged THA, or THA after failure of ORIF. This review was registered in PROSPERO (CRD42022329555).

Acute THA defined as early or primary THA, and surgeon used acute THA as the ultimate treatment for older patients with acetabular fracture in three weeks. In addition, the patients should not receive the any other operation before ultimate THA.

### Search strategy

According to the Cochrane Handbook for Systematic Reviews, we searched for the following terms in PubMed, Embase, and Cochrane Library: (“Arthroplasty, Replacement, Hip” OR “hip replacement” OR “hip arthroplasty”) AND (“acetabular fracture” OR “acetabulum fracture” OR “acetabul*”) AND (“old” OR “elderly” OR “elder” OR “geriatric”) The search timeline was from database construction to December 2021. There were no other restrictions on the search process.

### Information and data extraction

Two trained professional reviewers independently read the full text of documents that met the inclusion criteria and extracted the following information: study design, number of patients, age, sex, prosthesis, indications for THA, follow-up, and outcomes. The primary outcome was indications for THA, and the secondary item was the fracture type of patients receiving THA. The disagreements during this process were resolved by a third reviewer.

## Results

### Literature search process and results

**Figure 1:**
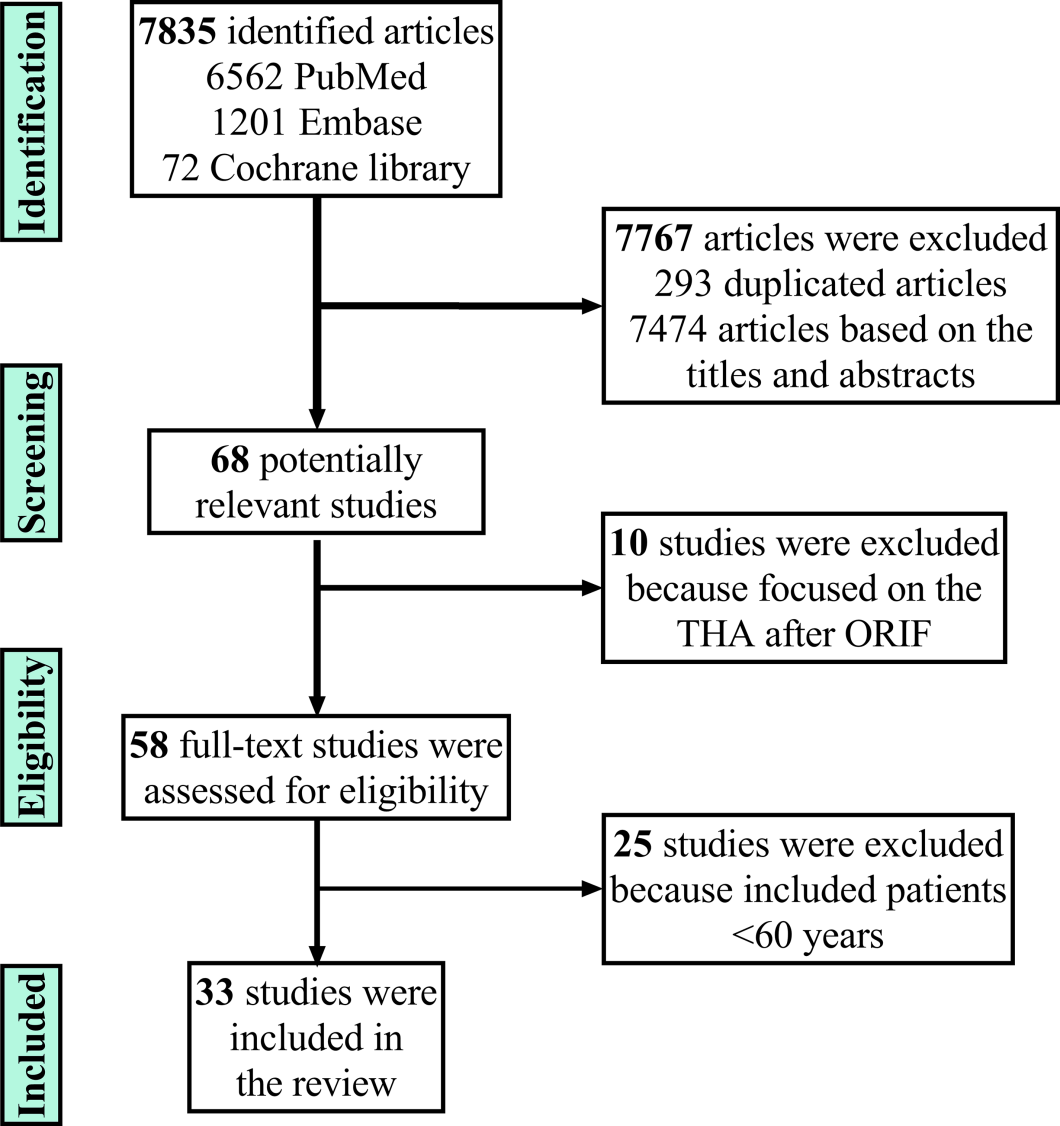
The flow of including studies.

Overall, 6562, 1201, and 72 papers (total: 7835) were obtained from Pubmed, Embase, and Cochrane Library, respectively. After excluding 293 duplicated papers, the reviewer read the title and abstract of each study and excluded 7,474 unrelated papers, leaving 68 studies. Most studies mentioned the revision operation after ORIF, and some studies included patients <60 years. After excluding these studies and studies without indicators of interest or fracture type, 33 studies were finally included (4, 6, 7, 11, 14–42). Figure 1 shows the flow chart of including studies.

### General information of the included study

**Figure 2:**
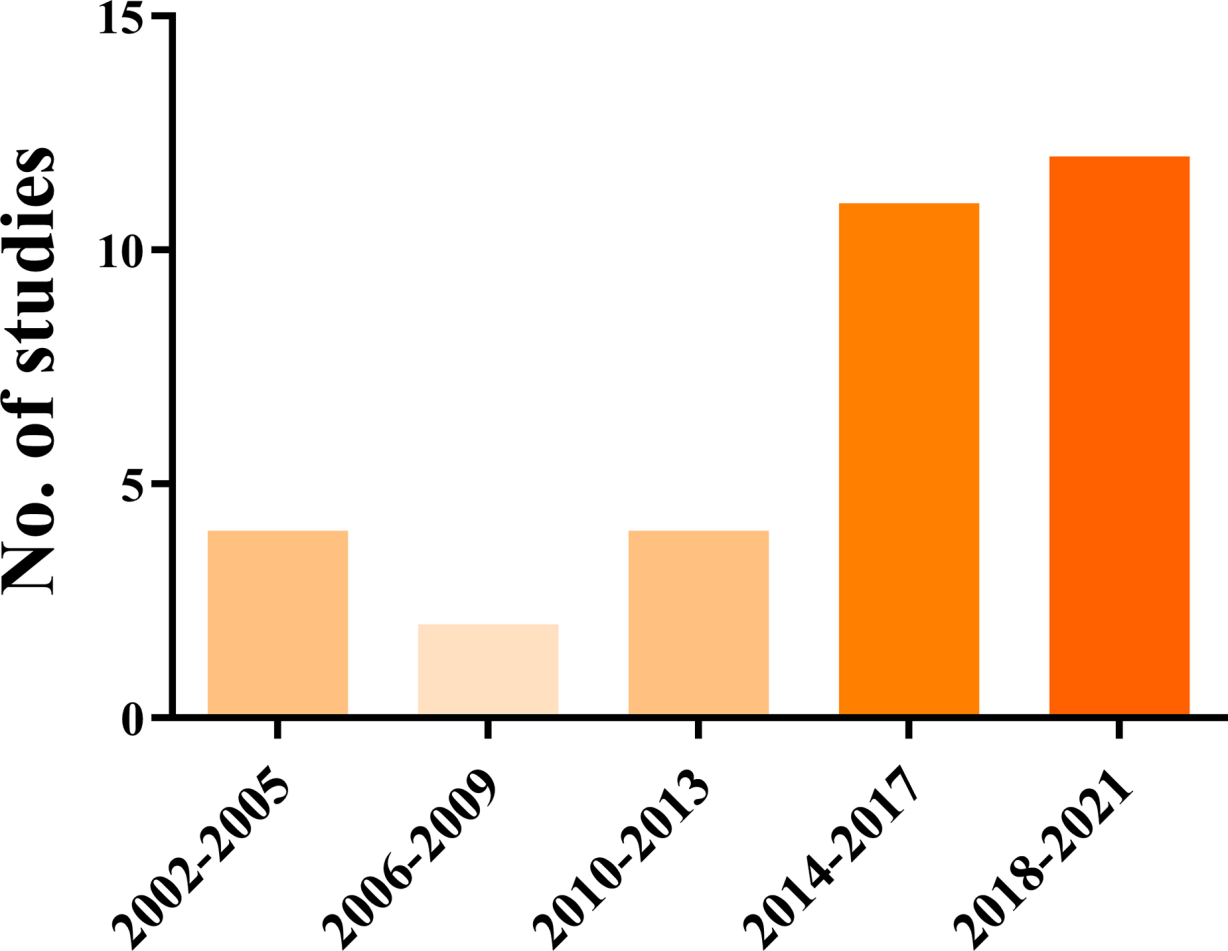
Change in number of studies with the year.

**Figure 3:**
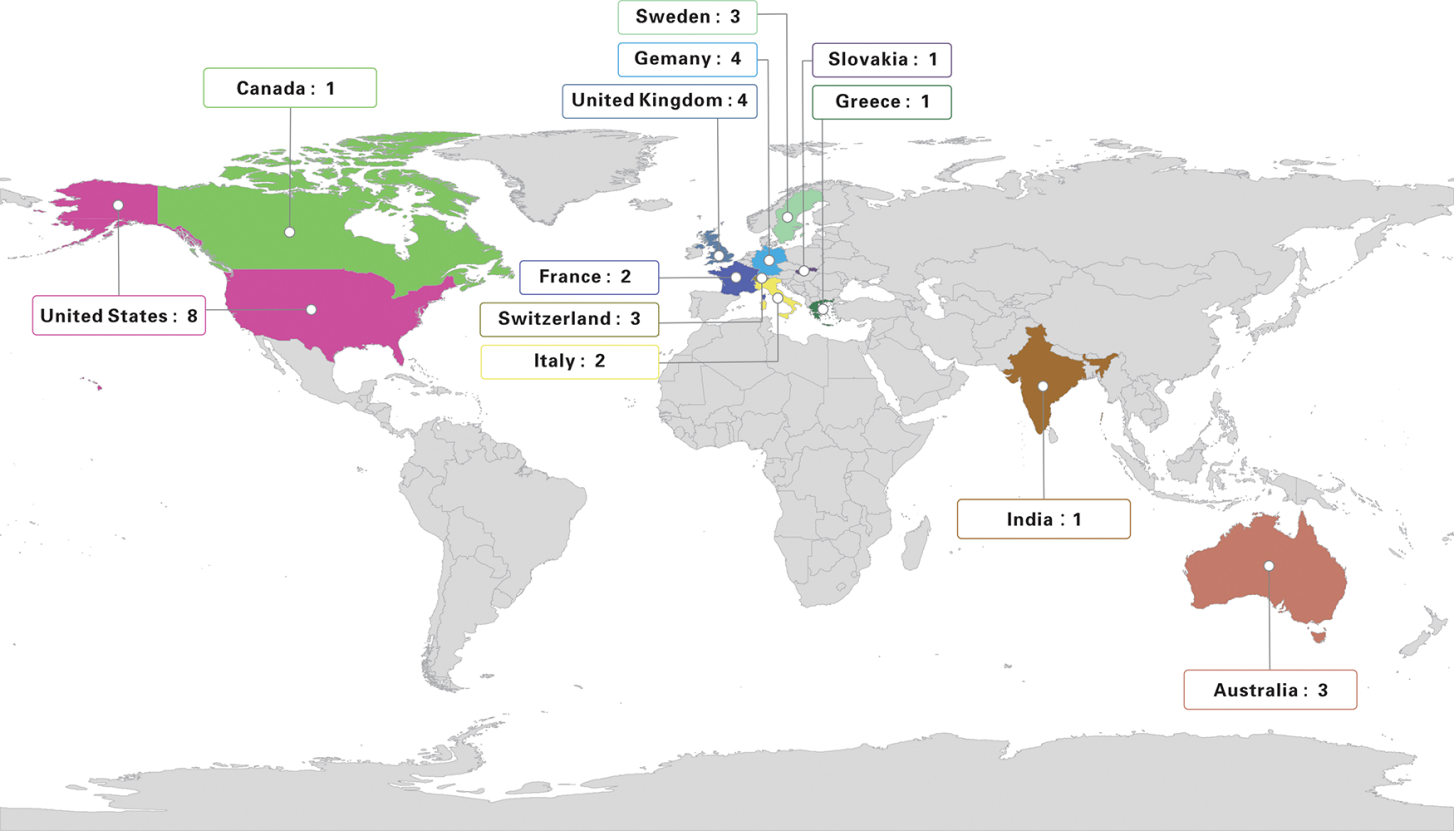
Included studies on the world map.

Of the 33 studies, 29 were published in English. These studies were published during 2002–2021. Figure 2 shows the change in number of studies with time. We found that in recent years, studies have focused on THA for older patients with acetabular fractures, especially from 2014 to 2021. In addition, eight studies were from the United States, four in Germany, four in the United Kingdom, three in Sweden, three in Switzerland, three in Australia, two in Italy, two in France, one in India, one in Canada, one in Greece, and one in Slovakia. We marked the included studies on the world map and noticed that majority of the research on THA for older patients were conducted in Europe and America (Figure 3).

### Main results

Of the 33 studies, 30 were retrospective and three were prospective. In total, we obtained 601 patients aged ≥60 years with acetabular fractures. In each study, the sample size was 6–55. Women accounted for 47.45% of all patients. The prosthesis type of THA fixation used were different across studies. Thirty-two studies reported follow-up, with the period ranging from 4.5 (7) to 81.5 months (40), of which 28 studies reported that THA or ORIF plus THA was a feasible treatment option for acetabular fractures in older patients. The remaining four did not identify the role of THA in older patients with acetabular fractures. Specific baseline information for the included studies is shown in Table 1.

**Table 1:** The baseline information for included studies.

Study	Design	No. of patients	Age	Female	Prosthesis	Indications for THA	Follow up (months)	Conclusions
Mouhsine 2002 (25)	Retrospective series	12	79 (65–93)	–	Press fit	Fracture involving weight bearing region, Dome impaction	24	THA provides good primary fixation, stabilizes complex acetabular fractures in elderly patients with osteoporotic bone and permits early postoperative mobilization.
Tidermark 2003 (30)	Retrospective series	9	75.2 (60–87)	3	Reinforcement ring	Fracture involving weight bearing region, Dome impaction, Low energy mechanism	38	THA seems to be a promising treatment alternative in displaced acetabular fractures in elderly patients with osteoporotic bone, except in those with an increased risk of dislocation
Borens 2004 (18)	Retrospective series	15	81	–	Press fit	Intra-articular impaction, Dome impaction	36	THA combined with internal fixation is a valid treatment option for acetabular fractures in the elderly.
Mouhsine 2004 (24)	Retrospective series	18	76 (65–93)	–	Press fit	Fracture involving weight bearing region, Dome impaction	36	THA provides good primary fixation, stabilizes complex acetabular fractures in elderly patients, and permits early postoperative mobilization
ŠIMKO 2006 (29)	Prospective series	10	71 (60–83)	6	Anti-protrusion cage	Irreducible articular comminution, Fracture involving weight bearing region, Pre-existing osteoarthritis or avascular necrosis, Femoral head injury	36	THA for acetabular fractures in elderly patients allows us to employ only one surgical technique for definitive repair.
Cochu 2007 (20)	Retrospective series	16	76.1 (64–89)	–	Reinforcement ring	Low energy mechanism	36	THA may provide several advantages including only one procedure and quick weight bearing with a lower rate of decubitus complications.
Herscovici 2010 (22)	Retrospective series	22	75.3 (60–95)	10	Press fit	Pre-existing osteoarthritis or avascular necrosis, Femoral head injury	29.4	The THA is an option for acetabular fractures in elderly patients.
Saxer 2011 (28)	Retrospective series	6	85 (82–89)	4	Press fit	Low energy mechanism	12	–
Rickman 2012 (27)	Retrospective series	12	75 (63–90)	3	Press fit	Low energy mechanism, Intra-articular impaction, Femoral head injury	18	Using a combination of acetabular fracture techniques and modern hip arthroplasty technology, it is possible to manage these patients in such a way as to allow immediate full weight bearing with very few complications.
Malhotra 2013 (40)	Retrospective series	12	66.1 (62–69)	1	Octopus ring	Femoral head injury, Femoral neck fracture, Irreducible articular comminution, Pre-existing osteoarthritis or avascular necrosis, Osteoporosis, Comorbidities	81.5	It is, therefore, worthwhile to recommend primary THA in the successful management of selected types of acetabular fractures in the elderly
Chakravarty 2014 (19)	Retrospective series	18	76.6 (61–90)	6	Press fit	Pre-existing osteoarthritis or avascular necrosis	22	This unique treatment of acetabular fractures has a role in carefully selected patients and provides the necessary reduction and immediate stability of the fracture
Enocson 2014 (21)	Retrospective series	15	75.5 (63–84)	7	Reinforcement ring	Fracture involving weight bearing region, Femoral head injury, Low energy mechanism	48	THA seems to be a safe option with good functional and radiologic outcomes
Rickman 2014 (4)	Retrospective series	24	77 (63–90)	8	Press fit	Comorbidities, Low energy mechanism, Irreducible articular comminution, Femoral head injury	6	Selected older patients with acetabular fractures may be managed using immediate weightbearing after fracture fixation and THA.
Solomon 2015 (14)	Retrospective series	11	81 (76–87)	7	Reinforcement ring	Low energy mechanism, Fracture involving anterior column, Irreducible articular comminution, Dome impaction	24	We continue to use THA routinely to treat patients with the same indications
Lin 2015 (38)	Retrospective series	23	72.3 (60–92)	14	Reflection cup/Restoration GAP II	Dome impaction, Irreducible articular comminution, Femoral head injury, Pre-existing osteoarthritis or avascular necrosis, Femoral neck fracture	67.2	Acute ORIF and immediate THA for selected acetabular fractures is a safe viable treatment option
Gary 2015 (36)	Retrospective controlled	36	77.1 (71–84)	16	–	–	12	–
Manson 2016 (23)	Retrospective controlled	20	>60	12	–	Female, Dome impaction, Low energy mechanism	–	–
Tissingh 2017 (11)	Retrospective cohort	19	77 (63–94)	9	Press fit	Dome impaction, Fracture involving anterior column, Femoral head injury, Fracture involving posterior wall/column	12	The result highlights the potential gains with THA
Ortega-Briones 2017 (26)	Retrospective series	24	77.4 (62–92)	4	Press fit	Low energy mechanism, Intra-articular impaction	49	Column fixation and simultaneous total hip arthroplasty are a viable option for complex geriatric acetabular fractures, with encouraging midterm results.
Boelch 2017 (7)	Retrospective controlled	9	79.8 (63–90)	4	Anti-protrusion cage	Irreducible articular comminution, Femoral head injury	4.5	Primary THA with an antiprotrusion cage should be strongly considered for osteoporotic acetabular fractures.
Weaver 2018 (6)	Retrospective controlled	37	79 (66–90)	18	Press fit/Anti-protrusion cage	High energy mechanism, Fracture involving anterior column	22	Acute reconstruction of acetabular fractures with THA in the geriatric population seems to compare favorably with ORIF.
Giunta 2019 (32)	Retrospective series	27	68.5 (60–84)	4	Reinforcement ring	Fracture involving posterior wall/column	48	Primary THA for acetabular fracture in the elderly population might be a good therapeutic option that allows return to the previous daily life activity.
Borg 2019 (16)	Prospective controlled	13	76.5 (64–89)	5	Burch-Schneider ring	Irreducible articular comminution, Dome impaction	24	The THA confers a considerably reduced need of further surgery
Lannes 2020 (31)	Retrospective controlled	26	78 (66–88)	11	Reinforcement ring	Irreducible articular comminution, Dome impaction, Femoral head injury, Femoral neck fracture, Osteoporosis, Pre-existing osteoarthritis or avascular necrosis	12	This study strengthens the practice of using only the posterior approach for primary THA in the elderly.
Liaw 2020 (33)	Retrospective series	20	73 (60–90)	3	Burch-Schneider cage	Irreducible articular comminution, Osteoporosis, Pre-existing osteoarthritis or avascular necrosis	26	This case series further validates the use of Burch-Schneider cages with primary THA in acute acetabular fractures.
Nicol 2020 (34)	Retrospective controlled	12	87 (68–93)	6	Cage	Comorbidities, Osteoporosis, Pre-existing osteoarthritis or avascular necrosis	60	In elderly acetabulum fractures, THA compared favorably with ORIF delayed THA
Sarantis 2020 (17)	Retrospective series	16	80.1 (76–89)	10	Reinforcement ring/antiprotrusion cage	Femoral head injury, Osteoporosis, Dome impaction, Irreducible articular comminution, Pre-existing osteoarthritis or avascular necrosis	72	Acute THA for the treatment of displaced acetabular fractures in elderly patients seems to be a safe option
Navarre 2020 (39)	Retrospective series	8	77 (61–89)	–	–	Femoral head injury, Dome impaction, Irreducible articular comminution	18	–
Aprato 2020 (15)	Retrospective series	55	71 (65–84)	16	Press fit	–	27	Good results in complex fracture patterns may also be achieved
Chen 2021 (35)	Retrospective series	7	82 (64–89)	2	Press fit	Irreducible articular comminution, Osteoporosis, Dome impaction	12	THA demonstrates satisfactory outcomes with low complications after one-year of follow-up
Manson 2021 (37)	Prospective randomized controlled trial	25	72.8	7	Press fit	Dome impaction, Femoral head fracture, Fracture involving posterior wall/column	12	Treatment with ORIF plus THA resulted in fewer reoperations than treatment with ORIF alone
Salama 2017 (41)	Retrospective series	14	71.2 (62–81)	5	Press fit	Irreducible articular comminution, Osteoporosis, Dome impaction, Femoral neck fracture, Pre-existing osteoarthritis or avascular necrosis	21	ORIF and simultaneous THR is a good option for the treatment of certain types of acetabular fractures particularly in elderly population.
Becker 2021 (42)	Retrospective series	10	74	4	Hip revision acetabular cup	Dome impaction	12	THA showed good results and is a feasible treatment option of acetabular fractures in geriatric patients

All the studies described their indications for THA in the patients, and the most reported indications were dome impaction (16★), irreducible articular comminution (13★), femoral head injury (12★), and pre-existing osteoarthritis or avascular necrosis (10★). All factors are summarized in Table 2.

**Table 2:** The main factors that should consider for THA.

Factors	No. of studies	No. of patients	Level
**Patient factors**			
Female	1	20	★
Comorbidities	3	48	★★★
Pre-existing osteoarthritis or avascular necrosis	10	173	★★★★★★★★★★
Osteoporosis	7	107	★★★★★★★
**Fracture factors**			
High energy mechanism	1	37	★
Low energy mechanism	9	137	★★★★★★★★★
Femoral head injury	12	196	★★★★★★★★★★★★
Femoral neck fracture	4	75	★★★★
Dome impaction	16	246	★★★★★★★★★★★★★★★★
Intra-articular impaction	3	51	★★★
Irreducible articular comminution	13	193	★★★★★★★★★★★★★
Fracture involving weight bearing region	5	64	★★★★★
Fracture involving anterior column	3	67	★★★
Fracture involving posterior wall/column	3	71	★★★

**Figure 4:**
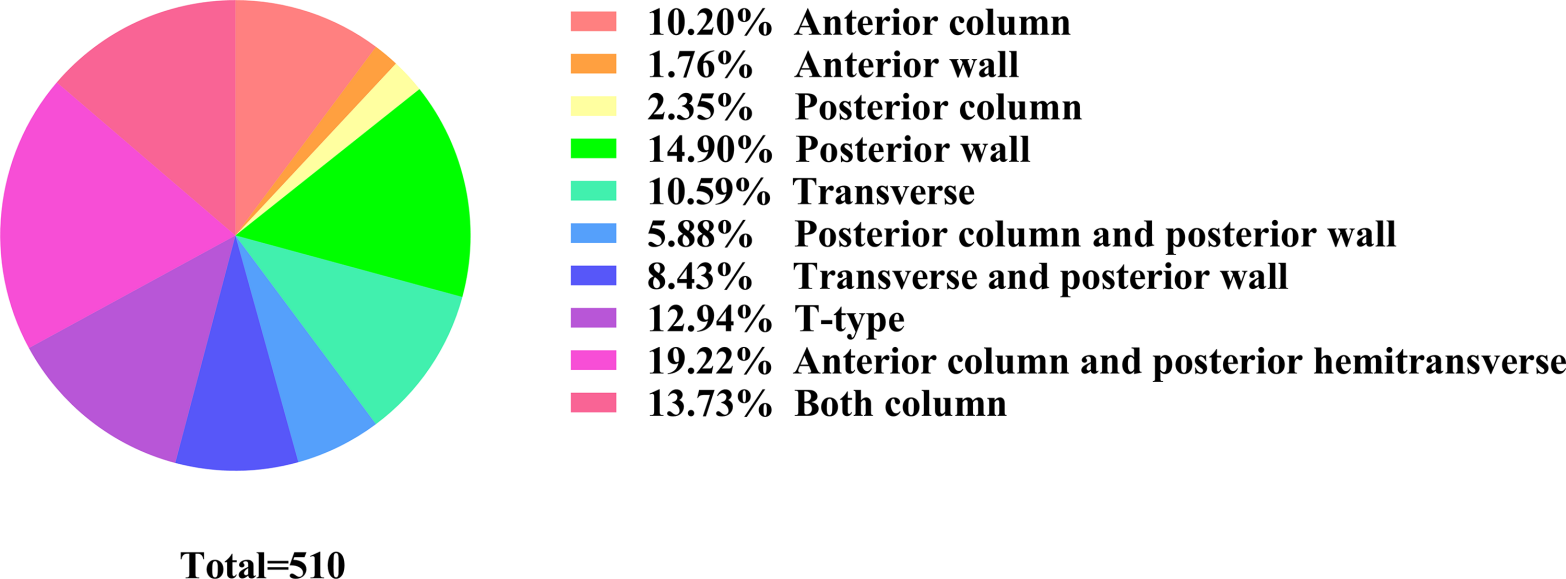
The percentages of ten fracture types in the studies.

As for fracture type, we have summarized the fracture types in Table 3. Detailed fracture type was reported for 510 patients. With respect to Letournel's classification system, 203 patients showed elementary patterns and 307 showed complex patterns. The most common patterns were anterior column and posterior hemitransverse (19.22%), posterior wall (14.90%), both columns (13.73%), and *T*-type (12.94%) (Figure 4).

**Table 3:** The fracture type in the studies.

Study	No. of patients	Anterior column	Anterior wall	Posterior column	Posterior wall	Transverse	Posterior column and posterior wall	Transverseand posterior wall	T-type	Anterior column and posterior hemitransverse	Both column
Boelch 2017 (7)	9	1	2	0	0	0	0	0	0	0	4
Weaver 2018 (6)	37	8	0	0	7	0	0	4	3	6	4
Manson 2016 (23)	20	3	0	1	4	0	4	1	1	3	3
Rickman 2012 (27)	12	2	0	0	0	0	2	1	5	1	1
Enocson 2014 (21)	15	10	0	0	0	1	0	0	0	4	0
Mouhsine 2002 (25)	12	0	0	0	0	0	4	0	8	0	0
Mouhsine 2004 (24)	18	0	0	0	0	2	2	4	9	1	0
Solomon 2015 (14)	11	1	0	0	0	0	0	0	0	7	3
Rickman 2014 (4)	24	4	0	0	1	8	3	0	0	2	6
Chakravarty 2014 (19)	18	1	0	0	0	3	1	3	1	5	4
Tidermark 2003 (30)	9	0	0	0	0	5	0	0	0	4	0
Herscovici 2010 (22)	22	0	0	0	0	0	0	9	0	7	6
ŠIMKO 2006 (29)	10	0	0	0	0	2	2	0	3	3	0
Saxer 2011 (28)	6	0	0	0	0	0	0	0	0	4	2
Cochu 2007 (20)	16	3	0	0	1	4	1	1	1	4	1
Borens 2004 (18)	15	0	0	2	0	2	0	2	8	0	1
Lannes 2020 (31)	26	1	0	0	3	5	1	2	3	6	5
Giunta 2019 (32)	27	0	0	2	9	4	0	0	2	5	5
Liaw 2020 (33)	20	2	0	0	1	0	1	0	3	5	8
Nicol 2020 (34)	12	1	0	0	0	0	1	0	2	5	3
Chen 2021 (35)	7	0	3	0	0	0	0	0	0	2	2
Gary 2015 (36)	36	4	2	1	5	4	0	3	3	6	2
Lin 2015 (38)	23	0	2	0	10	0	2	3	0	4	2
Sarantis 2020 (17)	16	9	0	0	0	2	0	0	0	5	0
Navarre 2020 (39)	8	1	0	0	2	1	1	0	0	1	2
Aprato 2020 (15)	55	1	0	4	21	9	0	6	12	2	0
Malhotra 2013 (40)	12	0	0	2	3	1	3	2	0	1	0
Salama 2017 (41)	14	0	0	0	8	1	1	1	2	0	1
Borg 2019 (16)	13	0	0	0	1	0	1	1	0	5	5
Total	510	52	9	12	76	54	30	43	66	98	70

## Discussion

In this review, we determined the most commonly reported indications for acetabular fractures, as well as the fracture patterns in older patients.

The incidence of acetabular fractures in older patients has rapidly increased worldwide. Most older patients have poor bone quality, multiple comorbidities, and increased perioperative risk, which may reduce the chance of favorable outcome (3, 43). Thus, surgeons should particularly note the characteristics of acetabular fractures in older patients and encourage early weight bearing. Although ORIF for acetabular fractures has gained wide acceptance in the treatment of young patients, the indications and results of this treatment in the older adult population remain unclear. On the one hand, the failure and conversion to THA rate in older patients is high after ORIF, which was reported as 19% by Archdeacon et al. (44), 25% by Gary et al. (45), 28% by O’Toole et al. (46), and 30.6% by Gary et al. (47); these are significantly higher than the 8.5% reported in the literature on the treatment of acetabular fractures in all age groups (48). On the other hand, the time point of THA was not long after ORIF, which was reported as within 2 years after ORIF in Weaver et al. (6), 19.6 months in Boelch et al. (7), a mean of 18 months in Archdeacon et al. (44), and a mean of 1.4 years after ORIF in Gary et al. (45). Of note, the survival of the native hip joint after ORIF was <2 years in most studies. Two or more operations within a short period for older patients with comorbidities is a serious concern. Therefore, appropriate patient selection is very important for ORIF, and THA should be considered as an initial alternative. Further, it is crucial to carefully select older patients with poor bone quality and multiple comorbidities as candidates for THA.

In this systematic review, all the studies described their indications for THA in the patients, and we give the image sign one star when the image sign was mentioned one time. By this way, the relative importance of image sign was identified. At last, we identified dome impaction, irreducible articular comminution, femoral head injury, and pre-existing osteoarthritis or avascular necrosis as the primary indications for THA in older patients with acetabular fractures. Dome impaction, the so-called “seagull sign,” is usually observed in osteoporotic and older patients. Anglen et al. reported that dome impaction was 100% predictive of ORIF failure in their series (12). However, in a study by Laflamme et al., after adequate reduction in the superomedial dome, the failure rate was 33% (49). Several studies reported osteoporosis as a commonly identified factor among the patients, but bone density was not regularly examined before treatment. Osteoporosis was speculated to cause low-energy fracture; osteoporotic patients with superomedial dome impaction did not benefit from attempted ORIF (12). Irreducible articular comminution was reported by 13 studies and is a subjective factor and varies in different-level surgeons. It was often followed by acetabular comminuted fracture or loss of bone and cartilage. In fact, we believe dome impaction and irreducible articular comminution are critical because they lead to difficult adequate reduction, especially in older patients with osteoporosis. For an identified fracture, dome impaction or articular comminution could be improved with surgical skill (50, 51), and good reduction of the articular surface can be achieved. However, the use of ORIF does not encourage early weight bearing. Femoral head injury and pre-existing hip degeneration were two important objective factors. Femoral head injury was reported in 10 articles, but the detailed injury model was not described and may involve fracture, cartilage contusion, or missing bone fragment. Pre-existing osteoarthritis or avascular necrosis was another important indication for THA. Thus, when any of these four indications are observed in preoperative images or during operation, THA or conversion to THA should be considered in operation.

As for the fracture type, the most patterns were the anterior column and posterior hemitransverse, posterior wall, both columns, and T-type, which contributed to >60% of all fractures. One elementary and three associated patterns were noted. Fractures of the posterior wall were the most common type of acetabular fractures (52), with a wide variety of fracture types (with respect to comminution, size and location of fragments, displacements, presence of marginal impaction, and labral avulsions) (53). Three types of associated patterns were often hard to reduce or fracture comminution. Failure to adequately deal with these types results in suboptimal reduction, inadequate fixation with recurrence of joint instability, and poor long-term results.

According to Rickman et al. (4), patients should be deemed sufficiently fit medically to undergo surgery. Although acute THA is a technically demanding intervention, without significant risks to the patient (11), other studies have not focused on all the factors reported in this study. To our knowledge, this is the first systematic review of these important factors. Indications for THA were based on a number of factors, and we included patient and fracture factors. Moreover, surgeon skill is another important factor. Different surgeons have different skill levels and experience in treatment of the fracture. Particularly, a difference in operation may be observed between senior and junior surgeons or between trauma and joint specialist surgeons.

This review has two limitations. Firstly, this is a systematic narrative review, not including typical meta-analysis because the included studies were series cases, and there is no controlled ORIF group. Therefore, there is no statistical analysis in forest plot, heterogeneity analysis, risk of bias and funnel chart. Secondly, all factors were obtained based on the frequency in studies; this method of assessment may not be accurate in identifying the most important indications. Surgeons must evaluate older patients with acetabular fractures before selecting the operation strategy. These factors may aid surgeons in selecting the optimal treatment option.

In conclusion, acute THA is an effective treatment strategy for older patients with acetabular fractures. We recommend THA when one of the signs of dome impaction, irreducible articular comminution, femoral head injury, and pre-existing osteoarthritis or avascular necrosis are present, especially in fracture patterns of the anterior column and posterior hemitransverse, posterior wall, both columns, and T-type.

## Data Availability

The original contributions presented in the study are included in the article/Supplementary Material, further inquiries can be directed to the corresponding author/s.
